# Orbital Apex Syndrome Following Herpes Zoster Ophthalmicus: A Case Report and Clinical Approach

**DOI:** 10.1002/ccr3.9710

**Published:** 2024-12-06

**Authors:** Mehrdad Motamed Shariati, Farid Shekarchian, Ali Zolfaghari

**Affiliations:** ^1^ Eye Research Center Mashhad University of Medical Sciences Mashhad Iran

**Keywords:** frozen eye, herpes zoster ophthalmicus, orbital apex syndrome, prognosis

## Abstract

Given the potential for rapid progression and significant morbidity, clinicians should maintain a high index of suspicion for OAS in patients with HZO who present with neurological symptoms such as ophthalmoplegia, ptosis, and sensory loss.

## 1 Introduction

Orbital apex syndrome (OAS) is an uncommon but significant clinical disorder that includes ophthalmoplegia, ptosis, visual loss, and sensory impairments in the ophthalmic branch of the trigeminal nerve [1]. These symptoms are caused by the involvement of many cranial nerves (III, IV, VI, and V1) and the optic nerve at the orbital apex, a vital anatomical juncture between the orbit and the cranial cavity. OAS has a wide range of causes, including neoplasia, trauma, vascular disease, inflammation, and infection [2]. Among infectious etiologies, herpes zoster ophthalmicus (HZO), albeit uncommon, contributes significantly to this condition [3].

HZO is caused by the reactivation of the varicella‐zoster virus (VZV) in the ophthalmic division of the trigeminal nerve, resulting in a painful, vesicular rash across the forehead, scalp, and periocular area. Although most HZO infections cause keratitis, uveitis, or other anterior segment complications, the virus can occasionally spread posteriorly, damaging the orbital apex and causing OAS [4]. The pathophysiology of OAS in patients with HZO is multifaceted, including direct viral invasion, subsequent inflammation, and potential vascular alterations affecting the nerves within the orbital apex. One rare consequence of patients with HZO is the development of OAS [5].

This case report highlights the clinical presentation, diagnostic challenges, and management strategies of a patient with OAS secondary to HZO.

## 2 Case History and Examination

**FIGURE 1:**
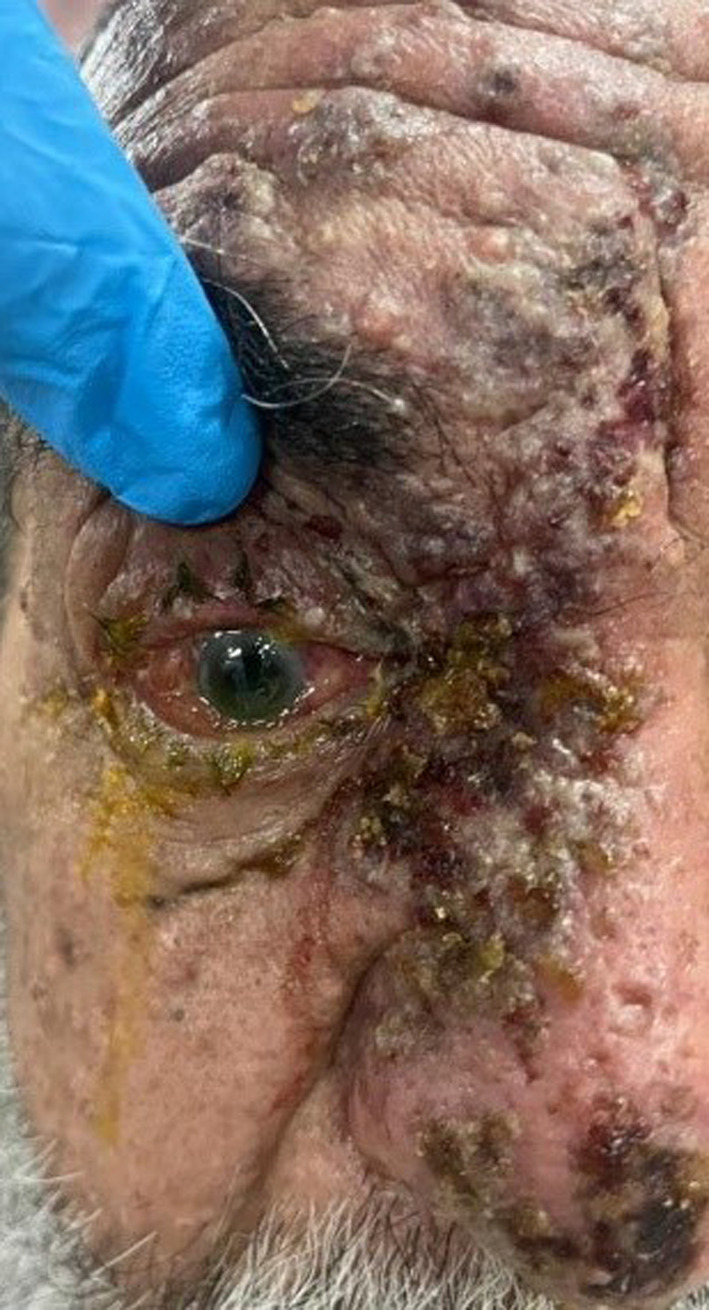
Eyelid swelling, mechanical ptosis, and vesicular lesions of the right eyelids and nose.

A 72‐year‐old man with a history of cataract surgery 1 month ago, presented with painful vesicular lesions of the right eyelids and nose (Figure 1). The patient's clinical symptoms started a week ago and had a progressive course. In the past medical history, the patient did not mention the history of specific diseases such as diabetes, autoimmune disorders, and immunodeficiency. The visual acuity was light perception and 10/10 for the right and left eyes. In the external examination, eyelid swelling, mechanical ptosis, and vesicular lesions were apparent. The relative afferent pupillary defect (RAPD) was strongly positive for the right eye (RE). The intraocular pressure was 13 and 15 mmHg. We found corneal punctate epithelial erosions in slit lamp examination of the RE. In the fundus examination, the optic nerve head was pale and edematous with indistinct margins. Ocular movement examination showed full limitation in all gaze directions in the RE. The left eye examination was unremarkable.

## 3 Diagnosis and Treatment

**FIGURE 2:**
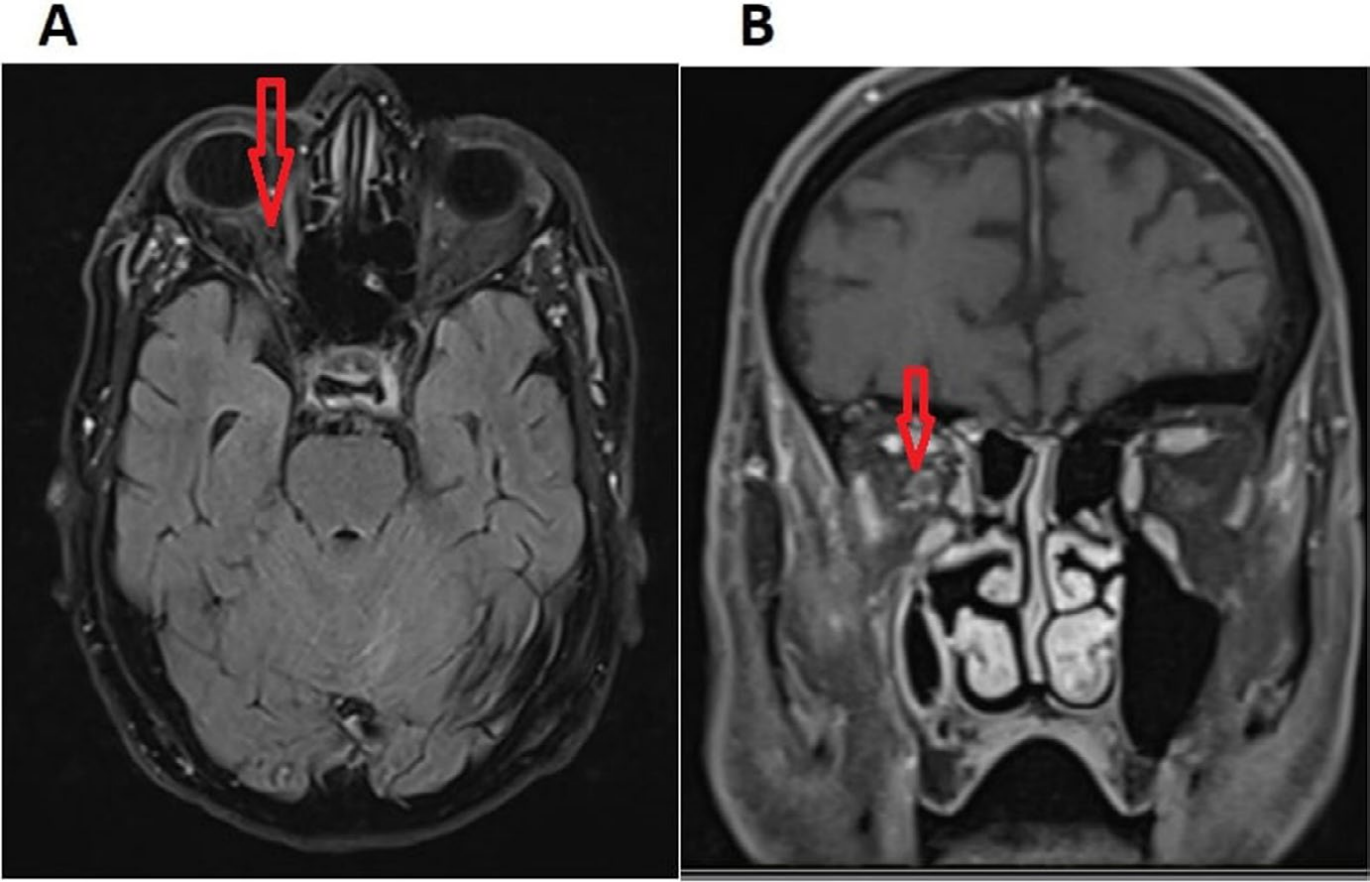
Axial (A) and coronal (B) T1 fat‐suppressed MRI scans with gadolinium enhancement show the orbital apex infiltration with the optic nerve sheath involvement of the right side (red arrow).

The OAS was considered regarding frozen eye and decreased visual acuity. Orbital and brain MRI with gadolinium enhancement was ordered. As we showed in Figure 2, the orbital apex infiltration was apparent in the MRI scans. The imaging findings (Figure 2) revealed no signs of mass effect or abnormal vascularity, which could suggest tumors or vascular lesions. Additionally, no inflammatory changes in adjacent sinuses were observed, helping to exclude sinus‐related inflammatory processes. These radiologic characteristics are consistent with HZO‐related OAS.

## 4 Conclusion and Results

**FIGURE 3:**
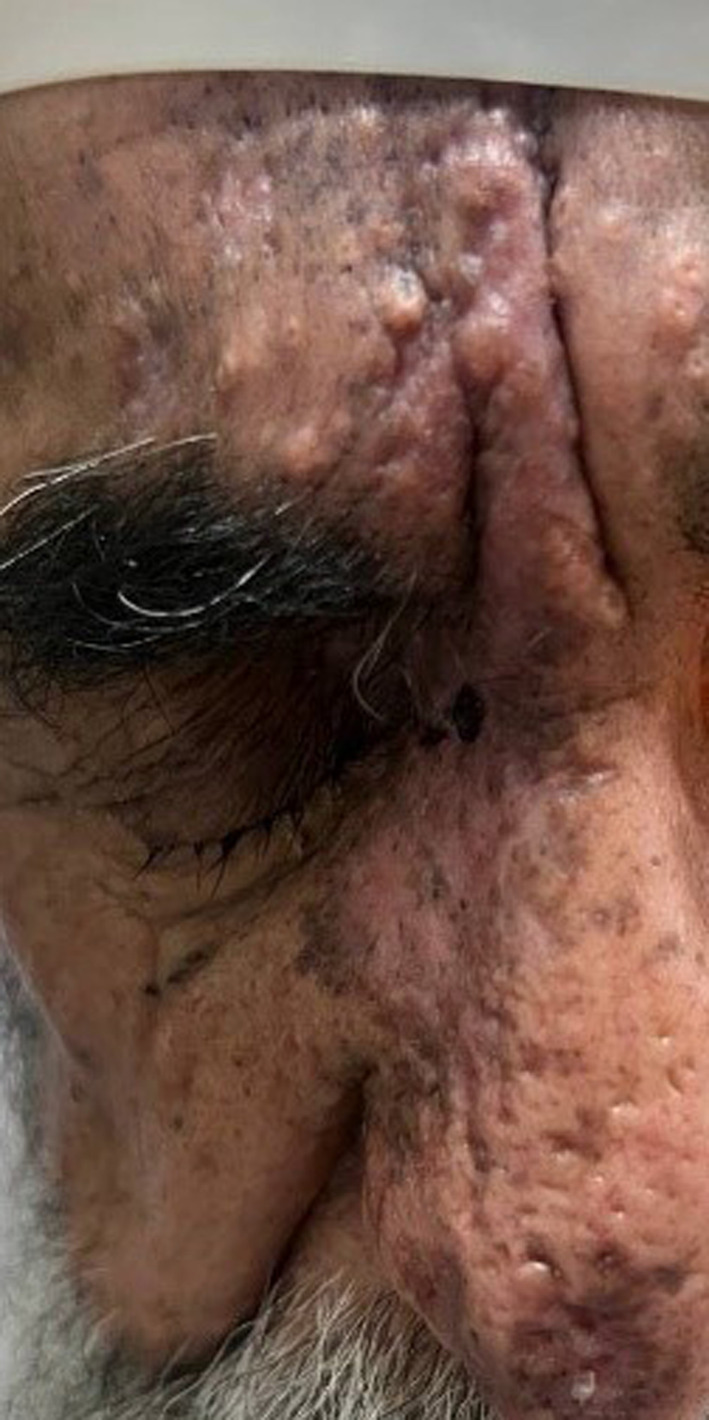
Regressed facial vesicular lesions.

The patient was hospitalized and treated with intravenous acyclovir, 500 mg every 8 h, and methylprednisolone, 250 mg every 6 h, for 1 week. The vesicular lesions regressed, and his visual acuity improved to counting fingers at 2.5 m (Figure 3).

The patient was discharged on a regimen of oral prednisolone at 0.5 mg/kg body weight and valacyclovir 1 g every 8 h. Corticosteroid was slowly tapered off over 1 month, and valacyclovir was continued for 12 weeks. At the one‐month follow‐up, ocular motility had improved significantly, with partial recovery of extraocular movements in the affected eye. Visual acuity in the RE had improved to counting fingers at 3 m. The optic nerve head appeared less edematous, though pallor persisted, suggesting some residual optic neuropathy. The vesicular skin lesions had resolved completely, and no new ocular or systemic complications were observed. Regular follow‐ups were planned to monitor for any delayed sequelae or recurrence.

## 5 Discussion

The mentioned patient is a case of rare but severe complications of HZO. Retro‐orbital optic nerve sheath enhancement was demonstrated by orbital MRI, which also ruled out other common causes of OAS, including cavernous sinus thrombosis, neoplasms, and hemorrhage. Zoster diagnosis is based on clinical appearance. The size of the vesicles is uniform in HSV and varied in VZV. Viral cultures, antigen detection, and molecular approaches are examples of laboratory tests that may accurately distinguish between these two conditions. However, in our case, the diagnosis was made on the basis of imaging results and clinical findings. OAS in HZO may have a complex etiology, including immune‐mediated inflammation, vasculopathy, and direct viral invasion. Perineural inflammation and viral dissemination down the ophthalmic division to the orbital apex might result from VZV reactivation inside of the trigeminal ganglion [6]. The virus may induce necrotizing inflammation, leading to the clinical signs of OAS by affecting the optic nerve, extraocular muscles, and other cranial nerves at the orbital apex. Furthermore, VZV prefers vascular tissues, which can result in vasculitis and impair the blood supply to the muscles surrounding the eyes and the optic nerve. This ischemic process might aggravate nerve injury [3, 5, 6]. In certain instances, the pathophysiology may be aided by an immune‐mediated mechanism that results from the inflammatory reaction to the virus. The combination of direct viral cytopathic effects, vasculitis‐induced ischemia, and immune‐mediated damage constitutes the foundation of OAS in HZO [7].

The clinical manifestation of OAS related to HZO contains the typical characteristics of both diseases. A painful vesicular rash in the V1 distribution characterizes HZO. As the disease advances to affect the orbital apex, cranial nerves III, IV, and VI become involved, resulting in symptoms such as diplopia, ptosis, and ophthalmoplegia. The incidence of extraocular muscle paralysis in patients with HZO has been estimated from 3.4% to 9.8%. The prevalence of optic nerve involvement is even lower and is 0.4% [4, 7]. Visual impairment may develop if the optic nerve is damaged, potentially leading to permanent vision loss if not treated immediately. Because of the involvement of the ophthalmic branch of the trigeminal nerve (V1), sensory impairments in the forehead and upper eyelid are also frequently seen [8, 9].

In this case, the patient had the classic vesicular rash of HZO, followed by a rapid visual impairment and ophthalmoplegia, consistent with OAS. This sequential process emphasizes the need to keep a high clinical suspicion for OAS in patients with HZO exhibiting neurological symptoms, as early detection and treatment are critical to preventing irreversible neuronal damage. According to previous studies, affected patients are usually over 60 years old. Our patient's age was compatible with past reports [4, 7].

**FIGURE 4:**
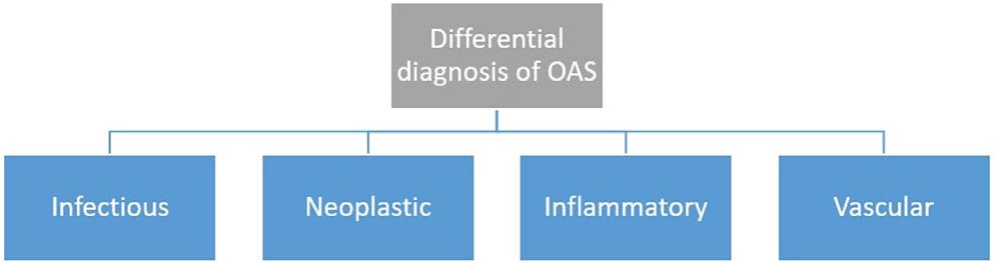
The main differential diagnoses of orbital apex syndrome (OAS).

Given the range of possible etiologies, the differential diagnosis for OAS is extensive. Differentiating between vascular, neoplastic, inflammatory, and viral causes is crucial (Figure 4; [1, 7]).

- *Infectious causes*: Aside from HZO, additional infectious causes of OAS include fungal infections (e.g., mucormycosis and aspergillosis), bacterial infections (e.g., orbital cellulitis), and viral infections (e.g., cytomegalovirus). However, a vesicular rash and a history of recent VZV reactivation might help narrow down the diagnosis to HZO [1].
- *Neoplastic causes*: The orbital apex may also be affected by tumors such as metastases, schwannomas, and meningiomas. Imaging tests, particularly MRI with contrast, are critical in distinguishing viral and inflammatory causes [1].
- *Inflammatory causes*: Granulomatosis with polyangiitis (previously known as Wegener's granulomatosis) and sarcoidosis are major inflammatory disorders that can affect the orbital apex. These disorders are frequently accompanied by systemic symptoms and biomarkers of inflammation [1].
- *Vascular causes*: Internal carotid artery aneurysms and cavernous sinus thrombosis might appear with similar symptoms. However, they frequently have a more abrupt onset and distinct risk factors, such as trauma or a hypercoagulable condition [1].

Given the patient's symptoms and the temporal relationship with HZO, alternative diagnoses were ruled out. Based on imaging and clinical data, OAS due to HZO was the first diagnosis.

The management of OAS in the context of HZO involves a combination of antiviral therapy, corticosteroids, and supportive care (Table 1). The primary goal of treatment is to reduce viral replication, control inflammation, and prevent further nerve damage [7, 10]. Considering that the optimal time to start treatment with acyclovir is in the first 72 h from the onset of symptoms, our patient probably showed a suboptimal response. Treatment regimen in patients with OAS in the context of HZO depends on the severity of inflammation and symptoms. In severe cases, it is recommended to start intravenous acyclovir treatment for 3–7 days, and then continue antiviral treatment for up to 12 weeks. However, in milder cases, oral antivirals can be started from the beginning. Previous studies showed inconsistent results regarding the duration of systemic corticosteroid therapy. A literature review shows that oral prednisolone at a dose of 0.5–1 mg/kg for 10–14 days can be reasonable and effective [4, 7].

**TABLE 1:** Recommended therapeutic measures in patients with OAS following HZO.

Antiviral therapy	High‐dose intravenous acyclovir or oral valacyclovir is the cornerstone of treatment. Early initiation of antiviral therapy is critical in reducing the severity and duration of symptoms and preventing complications such as OAS.
Corticosteroids	Systemic corticosteroids are often administered to reduce inflammation and edema at the orbital apex. This approach aims to minimize nerve damage and improve clinical outcomes. The dosing and duration of corticosteroids should be carefully monitored to balance the benefits against potential side effects, such as immunosuppression and the risk of secondary infections.
Pain management and supportive care	Neuropathic pain associated with HZO can be severe and debilitating. Adequate pain control with analgesics, including gabapentin or pregabalin, is necessary. In addition, supportive measures such as eye protection, lubricating eye drops, and management of any secondary complications, such as exposure keratitis, are essential components of care.
Ophthalmic interventions	Specific ophthalmic interventions may be required in cases with significant ocular involvement, including keratitis or uveitis, such as topical antivirals, corticosteroids, and pupil dilation to prevent synechiae.

The current case emphasizes the necessity of early detection and treatment of HZO to prevent its progression to OAS. The patient was treated with a combination of high‐dose antiviral medication and systemic corticosteroids, which improved symptoms but left some residual impairments.

The prognosis of OAS caused by HZO varies depending on the extent of nerve involvement and the timing of therapy. Prompt use of corticosteroids and antiviral medication often improves the prognosis of patients, whereas full symptom relief is not always possible. Residual impairments, notably in vision and ocular motion, are prevalent. Delayed diagnosis and treatment can result in irreversible optic nerve damage and persistent ophthalmoplegia, which can have a significant impact on the patient's quality of life. Hence, it is essential to uphold a high index of suspicion for OAS in individuals with HZO presenting neurological symptoms and to promptly start aggressive therapy at the onset of the disease [11, 12].

## 6 Conclusion

OAS secondary to HZO is a rare but serious condition that can lead to significant visual and functional impairments. Early diagnosis and prompt treatment with high‐dose antiviral therapy and corticosteroids are crucial to prevent disease progression and improve clinical outcomes. This case underscores the importance of recognizing HZO as a potential cause of OAS and initiating timely intervention to optimize recovery and minimize long‐term complications.

## Author Contributions

**Mehrdad Motamed Shariati:** conceptualization, data curation, visualization, writing – original draft, writing – review and editing. **Farid Shekarchian:** data curation, investigation, supervision, writing – review and editing. **Ali Zolfaghari:** data curation, visualization, writing – original draft.

## Ethics Statement

The authors have nothing to report.

## Consent

Written informed consent was obtained from the patient.

## Conflicts of Interest

The authors declare no conflicts of interest.

## Data Availability

The datasets used during the current study are available from the corresponding author upon reasonable request.
